# Adverse Events in Endoscopic Retrograde Cholangiopancreticography for Choledocholithiasis: A Holistic Perspective

**DOI:** 10.7759/cureus.53375

**Published:** 2024-02-01

**Authors:** Rasim Eren Cankurtaran, Osman Ersoy

**Affiliations:** 1 Department of Gastroenterology, Faculty of Medicine, Ankara Yildirim Beyazit University, Ankara, TUR; 2 Department of Gastroenterology, School of Medicine, Ankara Yildirim Beyazit University, Ankara, TUR

**Keywords:** interventional endoscopy, difficult cannulation, choledoccolithiasis, adverse events (aes), ercp

## Abstract

Background and aims

In this study, we aimed to investigate the frequency of adverse events (AEs) in patients undergoing endoscopic retrograde cholangiopancreticography (ERCP) for choledocholithiasis and the independent risk factors that may cause these conditions. We planned to evaluate all AEs including cardiopulmonary complications and the risk factors that may affect them holistically.

Methods

This study was designed as a retrospective cohort study conducted at a single tertiary center's gastroenterology clinic. The study included patients with naive papillae and undergoing ERCP for choledocholithiasis between May 2019 and June 2022. Risk factors that may lead to AEs were analyzed in terms of both patient-related factors and procedure-related factors. Patients with and without AEs after ERCP were compared.

Results

This study included 812 patients who underwent ERCP for choledocholithiasis. AE occurred in 149 (18.3%) of patients, and the most common complication was pancreatitis (n=112, 13.8%). In regression analysis, of the patient- and procedure-related factors, only difficult cannulation was a significant independent risk factor for AEs (odds ratio=3.85, 95% CI: 1.102-13.498, p=0.035).

Conclusion

This study showed that, of patient- and procedure-related factors, only difficult cannulation is an independent risk factor for ERCP-related AEs.

## Introduction

Endoscopic retrograde cholangiopancreaticography (ERCP) is an endoscopic procedure that has been used in the diagnosis and treatment of various pancreatobiliary diseases for more than 50 years and has become increasingly popular in recent years for its theropathic aspect [1,2]. Advancements in diagnostic imaging, cannulation techniques, and endoscopic accessories such as guidewires and stents have established ERCP as the leading treatment option for obstructive cholangitis, choledocholithiasis, and various other diseases [3].

Choledocholithiasis is a significant medical condition involving the presence of stones within the common bile duct, which warrants immediate intervention when bile drainage is highly compromised. Up to 20% of patients who exhibit symptoms of cholelithiasis have synchronous choledocholithiasis [4]. Choledocholithiasis can cause severe symptoms and complications in 1-2% of patients and is the most common indication for ERCP [5,6].

Over the past three decades, advances in ERCP have transformed the treatment of common bile duct stone removal from a major surgery to a minimally invasive procedure [4]. Despite the high success rate and effectiveness of ERCP in treating choledocholithiasis and other diseases, it is known that adverse events (AEs) may occur [7]. The prevalence and independent risk factors of ERCP-related AEs have been investigated in many studies in recent years [7-9]. Some studies have investigated the impact of procedure-related factors [10], and others have examined the impact of patient-related factors [11] on AEs. In these studies, factors influencing ERCP-related AEs such as pancreatitis, bleeding and perforation have generally been investigated, while studies investigating risk factors for all AEs, including cardiopulmonary complications, are relatively few [12].

The aim of this study was to investigate the incidence of AEs in patients undergoing ERCP for choledocholithiasis and the independent risk factors for these conditions, such as patient and procedure-related factors. We planned to evaluate all AEs including cardiopulmonary complications and the risk factors that may affect them holistically.

## Materials and methods

Patients and study design

This study was designed as a retrospective cohort study conducted at a single tertiary center's gastroenterology clinic. The study included patients with naive papillae and undergoing ERCP for choledocholithiasis between May 2019 and June 2022. Indications other than choledocholithiasis, patients with surgically altered anatomy (Billroth 2 gastrectomy or Roux-en-Y anastomosis), those under 18 years of age, those with any prior malignant disease, and those who had undergone sphincterotomy were excluded from the study. Patients with missing data and post-procedural hospitalization in another center were also excluded. Demographic, laboratory, and imaging data, ERCP findings, adverse events during and after ERCP, and clinical follow-up data of all patients in the study were analyzed from the electronic database and patient files.

ERCP procedures

ERCPs were performed using a lateral scope (TJF 190; Olympus Optical) by an endoscopist experienced in performing >800 therapeutic ERCPs annually. Sedation was administered intravenously by an anesthesiologist consisting of midazolam and propofol. Standard biliary cannulation using a guidewire and sphincterotome was performed initially. If selective biliary cannulation could not be achieved with this method, alternative techniques such as double guidewire and precut were used. In addition, the duration of cannulation and the total procedure time were recorded for all cases.

Definitions

Comorbidity scores were calculated for patients in the study using the Charlson comorbidity index (CCI) [13]. Successful cannulation was defined as the achievement of deep biliary cannulation. Some patients who failed to achieve deep biliary cannulation in the first session underwent repeat ERCP 48 hours later. Patients who failed to achieve deep biliary cannulation in the second session were defined as failed cannulation. Cannulation time was the duration between initially visualizing the papilla and deep cannulation. Total procedure time was the duration from the beginning to the end of the procedure. According to a recently published guideline, difficult cannulation was defined as failing to achieve biliary cannulation within five minutes or having two or more unintended pancreatic cannulations [14]. ERCP-related AEs in all patients following the procedure were defined based on the standards of international consensus [15]. Furthermore, cardiopulmonary complications, intensive care unit (ICU) admission, and mortality occurring during or after the procedure were defined as AE. In our study, cardiopulmonary complications were defined as hypotension, hypoxia, respiratory arrest, and cardiac arrest that occurred during the procedure and prevented the continuation of the procedure.

Objectives

The primary objectives of the study were to identify independent risk factors for AEs after ERCP. Risk factors that may lead to AEs were analyzed in terms of both patient-related factors, such as demographic data, comorbid diseases, comorbidity scores, anticoagulant/anti-aggregant medications and some laboratory data, and procedure-related factors such as cannulation success, difficult cannulation, cannulation time, total procedure time, and presence of periampullary diverticulum (PAD). The secondary objective of the study was to determine the frequency and distribution of AEs.

Ethical statement

Ethical approval was received from the local Ethics Committee (Date: 29.06.2022, approval number: E1-22-2706), and the study was conducted in accordance with the Declaration of Helsinki guidelines.

Statistical analysis

In our study, the data were analyzed using Statistical Product and Service Solutions (SPSS, version 25; IBM Corp., Armonk, NY). Mean, standard deviation, median (Q1-Q3), frequency, and percentage statistics were used to express the data. Chi-square (Pearson, Yates continuity correction, and Fisher's Exact) tests were used to analyze categorical variables. The Mann-Whitney U test was used to compare continuous variables according to complication status. For the variables considered to be risk factors according to complication status, first, a simple crude odds ratio (COR) was calculated, and then a multiple logistic regression analysis (adjusted odds ratio, AOR) was performed using the enter method for significant results. The significance level was defined as 0.05 for all tests.

## Results

Our study included 812 patients who underwent ERCP for choledocholithiasis whom 349 (43%) were male and the median age was 61 years (44.5-73.5). Table 1 shows the basic descriptive statistics of the patients included in the study.

**Table 1:** Baseline characteristics of the patients Continuous variables are presented mean ± standard deviation and categorical variables, n (%). HT: hypertension, DM: diabetes mellitus, COPD: chronic obstructive pulmonary disease, CKD: chronic kidney disease, Tbil: total bilirubin, GGT: gamma-glutamyl transferase, WBC: white blood count, CRP:c reactive protein; USG: ultrasonography

Patients	N (%)
Age	58.8 ± 18.6
Female gender	463 (57.0)
History of cholecystectomy	126 (15.5)
Charlson score	
≤2	463 (57.0)
2<	349 (43.0)
Comorbidities	
HT	348 (42.9)
DM	152 (18.7)
Cardiac diseases	136 (16.7)
COPD/ Asthma	43 (5.3)
CKD	26 (3.2)
Orthopedic diseases	16 (2.0)
Neurological diseases	47 (5.8)
Antiaggregant medication use	
No	671 (82.6)
Yes	141 (17.4)
Anticoagulant medication use	
No	786 (96.8)
Yes	26 (3.2)
Laboratory datas	
Tbil	3.55 ± 3.1
GGT	439.5 ± 351.6
WBC	9.22 ± 4.1
CRP	36.51 ± 52.3
Radiological datas	
Common bile duct dilatation on USG	500 (61.6)
Common bile duct stone or sludge	418 (51.5)

**Figure 1:**
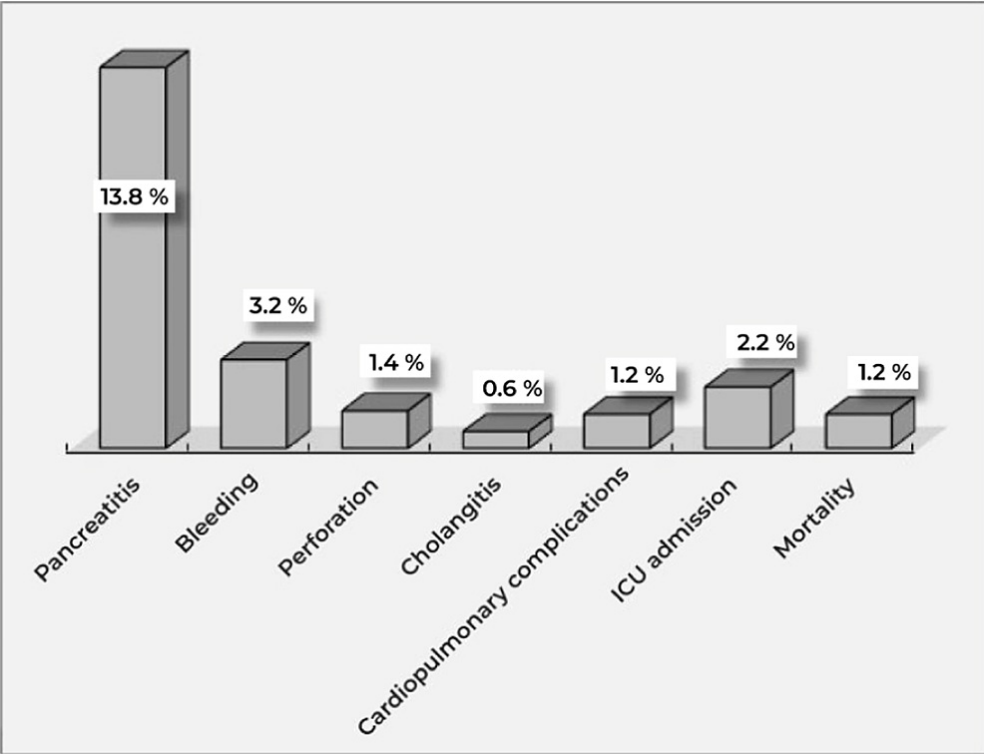
Frequency and distribution of ERCP-related adverse events

The frequency (18.3%) and distribution of ERCP-related AEs are shown in Figure 1. Pancreatitis (13.8%) was the most prevalent AE observed, whereas cholangitis (0.6%) was the least frequent.

Analysis of the patients' demographics, comorbidities, medications, and laboratory findings in relation to the occurrence of procedure-related AEs, as shown in Table 2, revealed a relatively homogeneous distribution, except for age, which approached significance (p=0.074). While most of the variables analyzed did not show statistical significance, the non-AE group's high-gamma-glutamyl transferase (GGT) values were significant (p=0.017).

**Table 2:** Comparison of variables associated with patients in terms of adverse events Continuous variables are presented in median (Q1-Q3) and categorical variables n (%). Mann-Whitney U, Pearson, Yates Continuity Correction, and Fisher's exact tests were used. HT: hypertension, DM: diabetes mellitus, COPD: chronic obstructive pulmonary disease, CKD: chronic kidney disease, Tbil: total bilirubin, GGT: gamma-glutamyl transferase, WBC: white blood count, CRP: c reactive protein

Variables	Non-adverse events	Adverse events	p
Age	60 (44-72)	64 (48-75)	0.074
Female gender	370 (79.1)	93 (20.1)	0.141
Charlson score			
≤2	382 (82.5)	81 (17.5)	0.468
2<	281 (80.5)	68 (19.5)	
History of cholecystectomy	103 (81.7)	23 (18.3)	0.976
Comorbidities			
HT	279 (80.2)	69 (19.8)	0.346
DM	124 (81.6)	28 (28.4)	0.980
Cardiac diseases	106 (77.9)	30 (22.1)	0.221
COPD/Asthma	37 (86)	6 (14)	0.574
CKD	19 (73.1)	7 (26.9)	0.299
Orthopedic diseases	15 (93.8)	1 (6.3)	0.329
Neurological diseases	41 (87.2)	6 (12.8)	0.409
Anti-aggregant medication use			
No	552 (82.3)	119 (17.7)	0.323
Yes	111 (78.7)	30 (21.3)	
Anticoagulant medication use			
No	643 (81.8)	143 (18.2)	0.605
Yes	20 (76.9)	6 (23.1)	
Laboratory data			
Tbil	2.8 (1.3-4.9)	2.6 (1-4.4)	0.224
GGT	371 (197-633)	333 (116-548)	0.017
WBC	8.32 (6.5-11.2)	8.35 (6.5-11.5)	0.996
CRP	12.5 (4.7-47.2)	13.8 (4.4-40.4)	0.787

When the procedure-related findings in patients with AEs were evaluated (Table 3), it was concluded that 34% of difficult cannulation was seen in the AE group (p<0.001). In addition, cannulation time (p<0.001) and total procedure time (p<0.001) were significantly higher in the AE group. In cannulation techniques, wire-guided cannulation (85.5%) and double guidewire (70%) techniques were frequently involved in the absence of post-ERCP AEs, whereas almost half (48.3%) of the patients using the precut technique developed AEs (p<0.001). In addition, a fully covered metal stent placement procedure was significantly higher in patients with AEs (p<0.001).

**Table 3:** Comparison of variables associated with procedures in terms of adverse events Continuous variables are presented in median (Q1-Q3) and categorical variables, n(%). Mann-Whitney U, Pearson, Yates Continuity Correction, and Fisher's exact tests were used. PAD: periampullary diverticulum, EBD: endoscopic biliary dilatation

Variables	Non-adverse events	Adverse events	p
Successful cannulation in the first session	642 (82.2)	139 (17.8)	0.771
Overall successful cannulation	659 (81.7)	148 (18.3)	-
Difficult cannulation	141 (66.2)	72 (33.8)	<0.001
Cannulation time (minutes)			
<5	526 (86.8)	80 (13.2)	<0.001
5-10	65 (67)	32 (33)	
10<	65 (67)	32 (33)	
Total procedure time (minutes)	25 (21-28)	28 (24-35.5)	<0.001
Presence of PAD	82 (79.6)	21 (21.4)	0.663
Cannulation tecnique			
Wire-guided cannulation	550 (85.5)	93 (14.5)	<0.001
Double guidewire tecnique	98 (70)	42 (30)	
Precut tecniques	15 (51.7)	14 (48.3)	
Sphincterotomy	640 (81.7)	143 (18.3)	0.740
Stone removal (Balloon or basket)	490 (80.7)	117 (19.3)	0.241
Plastic stent placement	369 (80.4)	90 (19.6)	0.291
Covered metal stent placement	1 (7.7)	12 (92.3)	<0.001
EBD	25 (83.3)	5 (16.7)	0.865

Single logistic regression analysis according to post-ERCP AE status revealed that elevated GGT and successful cannulation in the first session variables (very low level for GGT) played a protective role (COR=0.99, CI: 0.90-0.99, p=0.037 and COR=0.45, CI: 0.21-0.99, p=0.046, respectively). However, we found that difficult cannulation increased the risk of AE approximately 3.5-fold (p<0.001), cannulation time between 5-10 min increased the risk of AE approximately 3.2-fold (p<0.001), and cannulation time over 10 min increased the risk of AE approximately 3.5-fold (p<0.001). It was also concluded that the total procedure time increased the risk of AE approximately 1.1-fold (p<0.001), double guidewire technique 2.5-fold (p<0.001), and precut technique 5.5-fold (p<0.001). When the variables determined to be significant in univariable analysis were then evaluated by multiple logistic regression, it was found that only difficult cannulation increased the risk of complications in the presence of other variables (AOR=3.857, CI: 1.102-13.498, p=0.035) (Table 4).

**Table 4:** Univariable and multivariable analysis according to ERCP-related AE HT: hypertension, DM: diabetes mellitus, COPD: chronic obstructive pulmonary disease, CKD: chronic kidney disease, Tbil: total bilirubin, GGT: gamma-glutamyl transferase, WBC: white blood count, CRP: c reactive protein

Variables	Univariable analysis			Multivariable analysis		
Female gender	1.315	0.913-1.895	0.142			
Charlson score	1.141	0.798-1.631	0.469			
History of cholecystectomy	0.992	0.607-1.623	0.976			
HT	1.187	0.831-1.696	0.346			
DM	1.006	0.638-1.585	0.980			
Cardiac diseases	1.325	0.844-2.080	0.222			
COPD/Asthma	0.710	0.294-1.714	0.446			
CKD	1.671	0.689-4.050	0.256			
Orthopedic diseases	0.291	0.038-2.224	0.234			
Neurological diseases	0.637	0.265-1.538	0.312			
Anti-aggregant medication use	1.254	0.800-1.965	0.324			
Anticoagulant medication use	1.349	0.532-3.419	0.528			
TBil	0.990	0.934-1.051	0.752			
GGT	0.999	0.990-0.999	0.037	0.999	0.999-1.00	0.084
WBC	1.010	0.963-1.040	0.958			
CRP	1.001	0.997-1.004	0.707			
Successful cannulation in the first session	0.455	0.209-0.987	0.046	1.461	0.531-4.020	0.463
Difficult cannulation	3.462	2.388-5.018	<0.001	3.857	1.102-13.498	0.035
Cannulation time (minutes)						
5-10	3.237	1.995-5.253	<0.001	0.981	0.279-3.454	0.976
10<	3.540	2.205-5.684	<0.001	0.775	0.203-2.954	0.709
Total procedure time	1.054	1.032-1.076	<0.001	1.020	0.984-1.057	0.284
Presence of PAD	1.162	0.694-1.948	0.568			
Double guidewire	2.535	1.660-3.869	<0.001	0.740	0.351-1.552	0.426
Precut	5.520	2.579-11.812	<0.001	1.549	0.537-4.462	0.418

## Discussion

This study demonstrated that difficult cannulation is an independent risk factor associated with an increase in AEs in patients undergoing ERCP for choledocholithiasis. The study showed that none of the patient-related factors had a significant effect on AEs.

The frequency and risk factors of ERCP-related AEs have been the subject of many studies in recent years. In a multicenter study examining post-ERCP complications and predictive factors, the frequency of AEs after ERCP was found to be 11.6% among all patients (the most common complication was cholangitis with 3.8%) [16]. In a recently published study including 620 patients, the overall complication rate after ERCP was 11.8%, and the most common complication was pancreatitis with 8.2% [17]. In our study, AE occurred in 149 (18.3%) of patients, and the most common complication was pancreatitis (n=112, 13.8%). It is seen that the frequency of AEs in our study is higher compared to the above studies. The fact that the frequency of PEP was slightly higher may be considered to be effective in this result. PEP is known to be the most common AE associated with ERCP [18]. In a meta-analysis of data from many different countries, the frequency of PEP was reported to be between 8.4 and 14.7% [19]. In this study, the frequency of PEP was in a similar range to the rates in the meta-analysis. Among the patients with PEP, three patients had severe pancreatitis, and six patients had moderate pancreatitis, while all the remaining patients had mild pancreatitis. The rate of bleeding, another serious cause of AE in our study, was 3.2%. There are publications in the literature suggesting that bleeding may occur in a wide range of 0.3-9.6 [19]. In the American Society for Gastrointestinal Endoscopy (ASGE) guidelines, bleeding rates were reported to be between 0.3% and 2% [18]. All except three of our patients with bleeding experienced mild and intraprocedural symptoms. In the other patients, delayed moderate bleeding occurred and a fully covered metal stent was implanted. In addition, the rates of perforation and cholangitis were close to the literature [18].

In recent years, sedation-related complications, such as cardiopulmonary complications, have been included in guidelines as ERCP-related AEs [18,20]. Since ERCP is a sedation-assisted procedure, we think that cardiopulmonary complications should not be evaluated separately from other complications. Although there are efforts to standardize the methods of reporting cardiopulmonary AEs with endoscopy, the number of studies including these definitions is small [21]. Publications indicating that cardiopulmonary complications are between 4% and 16% according to the definitions used are available in the literature [22]. However, in studies excluding transient hypoxia and transient hypotensive states during the procedure, the rate of clinically significant cardiopulmonary AE ranges from 0.04% to 2.4% [16,23]. Significant cardiopulmonary complications in our study are similar to these rates. In this study, mortality occurred secondary to cardiopulmonary complications during or after the procedure in all but two patients. The other two patients had mortality secondary to sepsis after prolonged ICU hospitalization after perforation. In a nationally based study, it was reported that postoperative death occurred in 1.97% of 8,790 patients who underwent ERCP for choledocolithiasis [24]. These data suggest that our mortality data are at an acceptable rate.

The other main results of our study are the independent risk factors affecting AE. Guidelines published on ERCP-related AEs have specified some independent risk factors related to the patient and procedure [18,20]. However, in these guidelines, each risk factor was analyzed within its own complications, and risk factors related to sedation-related cardiopulmonary complications were not evaluated.

Patient-related risk factors for AE after ERCP have been the subject of many studies in recent years. It has been reported in the ASGE guideline that ages below 30 years and above 70 years are risk factors for PEP [18]. The European Society of Gastrointestinal Endoscopy (ESGE) guideline suggested being over 60 years of age as a risk factor for cholangitis [20]. Female gender was reported to be a risk factor for PEP by both guidelines. Studies examining the effect of comorbid diseases on ERCP results are available in the literature. A nationally based study found that the frequency of AEs after ERCP was significantly higher in patients with end-stage renal disease [11]. There are also publications indicating that anticoagulant use increases the risk of bleeding. In these publications, anticoagulant treatment within three days was reported to be risky in terms of bleeding [25]. In our study, antiaggregant and anticoagulant use was not found to be an independent risk factor for AE. Except for the patients who did not require emergency ERCP, all of the patients had discontinued their medications five days in advance and were receiving low molecular weight heparin as bridge therapy. Sphincterotomy was not performed in patients who underwent emergency ERCP and whose medications could not be discontinued. In addition, none of the patient-related factors such as age, gender, comorbid diseases, drug use, and laboratory findings significantly affected the risk of AE in our study.

It has also been suggested that there are many risk factors related to procedure-related factors affecting AE. In a retrospective study conducted to develop an online app to predict AEs after ERCP, sphincterotomy, ERCP with grade 3 and 4 complexity, and metal stent placement were found to be procedural risk factors for AEs. This publication is similar to our study in terms of evaluating AE risk factors holistically [12]. In another study of 2,800 ERCP procedures, precut sphincterotomy was suggested to be a risk factor for serious AE [16]. There are publications in the literature indicating that difficult cannulation, length of total procedure time, and double guidewire cannulation are also risk factors for PEP [7,18]. In this study, we found that only difficult cannulation was an independent risk factor for AE. Although total procedure time and length of cannulation time, precut techniques, and metal stent placement were found to be significantly higher in univariate analysis, they were not found to be significant in multivariate analysis.

The retrospective and single-center nature of the study constituted the most important limiting aspect of the study. In addition, although single-operator data were included in our study to exclude operator-related variability, this can be considered a limiting factor of the study.

## Conclusions

Our study holistically evaluated all AEs occurring in patients undergoing ERCP for choledocholithiasis. This study showed that, of patient- and procedure-related factors, only difficult cannulation is an independent risk factor for ERCP-related AEs.
